# Investigation of the Effects of Methylphenidate on Hemodynamic Parameters in Children With ADHD

**DOI:** 10.1002/prp2.70276

**Published:** 2026-06-04

**Authors:** Caglar Charles Daniel Jaicks, Selman Vefa Yıldırım

**Affiliations:** ^1^ Department of Child and Adolescent Psychiatry Istanbul Aydin University Istanbul Turkey; ^2^ Department of Pediatric Cardiology Near East University Nicosia Cyprus

**Keywords:** ADHD, electrocardiography, hemodynamics, methylphenidate

## Abstract

This prospective observational study investigated the acute effects of a single 10 mg dose of methylphenidate (MPH) on hemodynamic and electrocardiographic parameters in 60 children aged 6–16 years newly diagnosed with Attention Deficit Hyperactivity Disorder (ADHD). All participants received 10 mg short‐acting MPH, and systolic and diastolic blood pressure, heart rate, and electrocardiographic measurements (QTc, PR, QRS intervals, and rhythm) were obtained both before and 30–45 min after administration under standardized conditions and interpreted by a pediatric cardiologist. Following MPH intake, significant increases were observed in systolic blood pressure (105.0 ± 7.7 to 110.1 ± 8.9 mmHg, *p* < 0.001), diastolic blood pressure (69.3 ± 9.2 to 73.4 ± 9.8 mmHg, *p* < 0.001), and heart rate (79.8 ± 9.0 to 84.9 ± 9.8 bpm, *p* < 0.001). QTc (395.9 ± 11.6 to 401.0 ± 12.5 ms), PR (135.8 ± 16.8 to 140.9 ± 16.7 ms), and QRS durations (91.1 ± 9.5 to 93.6 ± 9.5 ms) also showed significant prolongation (all *p* < 0.001). Post‐treatment ECG rhythm abnormalities were observed in 15.0% of participants (*p* = 0.002), including sinus tachycardia, sinus arrhythmia, and isolated premature atrial or ventricular beats, with markedly higher prevalence among those below the 5th BMI percentile (75.0%, *p* < 0.001), whereas no rhythm disturbances were detected in the 5th–85th percentile group. These findings indicate that MPH induces acute but measurable cardiovascular changes in children with ADHD, particularly in underweight children, underscoring the need for ECG and blood pressure monitoring during treatment initiation.

## 1 Introduction

Attention Deficit Hyperactivity Disorder (ADHD) is a common neurodevelopmental disorder in childhood, characterized by symptoms such as reduced attention span, impulsivity, and hyperactivity [1]. The etiology of ADHD is multifactorial, and it is thought to result from the interaction of genetic, environmental, and neurobiological factors. The disorder can adversely affect an individual's academic performance, social relationships, and quality of life. Therefore, early diagnosis and effective treatment are of great importance [2, 3].

In the treatment of ADHD, pharmacological interventions are frequently preferred in addition to behavioral approaches [4]. Among central nervous system stimulants, methylphenidate is widely used as one of the first‐line medications for ADHD treatment. Methylphenidate exerts its effects by inhibiting the reuptake of dopamine and norepinephrine, thereby regulating attention, motivation, and executive functions in the prefrontal cortex [5]. The clinical efficacy of this drug has been supported by numerous studies, and when evaluated in terms of positive treatment response rates, it demonstrates high success rates in the child and adolescent population [6].

However, the potential effects of methylphenidate on the cardiovascular system have become an important area of research in recent years [7]. There is evidence suggesting that the use of methylphenidate may lead to changes in heart rate, blood pressure, and other hemodynamic parameters [8]. Therefore, it is emphasized that particular caution should be exercised during treatment, especially in individuals with underlying cardiovascular disease. Nevertheless, the clinical significance and persistence of these effects remain subjects of debate in the literature, with varying results reported.

The aim of this study is to evaluate the acute hemodynamic and electrophysiological effects of a single standardized 10 mg dose of methylphenidate in children newly diagnosed with ADHD. Unlike previous studies that primarily focus on long‐term treatment or heterogeneous dosing regimens, this study specifically assesses the peak‐effect period (30–45 min) following initial administration. In addition, the study examines whether BMI percentile groups exhibit differential cardiovascular responses, a question that has not been adequately addressed in existing literature. By comparing pre‐ and post‐treatment measurements of heart rate, blood pressure, and electrocardiographic parameters, this study aims to identify potential early cardiovascular changes and provide clinically relevant insights to support risk‐stratified monitoring during methylphenidate initiation.

## 2 Material and Methods

### 2.1 Study Design and Participants

This prospective, observational study was conducted at the child and adolescent psychiatry outpatient clinic of Medical Park Seyhan Hospital, Adana, Turkey. Ethical approval was obtained from the Non‐Interventional Clinical Research Ethics Committee of Çukurova University (Meeting No: 138, Decision No: 59, Date: November 3, 2023). Written informed consent was obtained from parents or legal guardians, and assent was obtained from children when appropriate. Children aged 6–16 years who were newly diagnosed with Attention Deficit Hyperactivity Disorder (ADHD) according to DSM‐5 criteria and were to be started on methylphenidate treatment were included. Exclusion criteria included: (1) known congenital or acquired cardiovascular disease; (2) arrhythmias previously documented on ECG or Holter monitoring; (3) chronic systemic illness (e.g., renal, hepatic, endocrine disorders); (4) the use of medications affecting blood pressure, heart rate, or cardiac conduction; (5) acute infection or fever at the time of evaluation; (6) electrolyte imbalance; and (7) inability to remain still during ECG acquisition. Children with incomplete clinical data or poor‐quality ECG recordings were also excluded. During screening, 8 patients were excluded due to incomplete clinical data (*n* = 3), poor‐quality ECG recordings (*n* = 3), and the use of medications affecting blood pressure, heart rate, or cardiac conduction (*n* = 2). Consequently, 60 participants met all eligibility criteria and completed both pre‐treatment and post‐treatment assessments.

### 2.2 Treatment Protocol

All eligible participants received a single 10 mg dose of standard short‐acting methylphenidate hydrochloride (Ritalin) in the clinic. International guidelines (AAP, AACAP, NICE) recommend initiating short‐acting methylphenidate within the 5–10 mg range, and since a 5 mg formulation is not available in Turkey, a 10 mg dose represents the lowest clinically feasible and guideline‐concordant starting option [9]. For the purposes of methodological standardization in this study, all participants received the same 10 mg dose. If no adverse reactions occurred, patients were subsequently transitioned to long‐acting preparations as clinically indicated.

### 2.3 Measurement of Hemodynamic Parameters

Hemodynamic parameters, including heart rate, systolic blood pressure (SBP), and diastolic blood pressure (DBP), were measured for each participant. All vital signs and blood pressure measurements were performed manually by the same pediatric nurse working in the child and adolescent psychiatry clinic, using the same calibrated pediatric sphygmomanometer and stethoscope, under standardized conditions. Measurements were taken in a quiet environment, with the child in a seated position and the right arm at heart level, following a rest period of 10 min. The average of two consecutive readings was used for analysis. A standard 12‐lead digital electrocardiogram (ECG) was obtained from each participant both before and 30–45 min after methylphenidate administration, corresponding to the expected peak plasma effect of the short‐acting formulation. All ECGs were recorded using a calibrated digital device at a paper speed of 25 mm/s and an amplitude of 10 mm/mV, with the child resting in the supine position for at least 10 min in a quiet, temperature‐controlled room. Heart rate, PR interval, QRS duration, QT interval, and corrected QT interval (QTc) were measured and manually verified by S.V.Y., a board‐certified pediatric cardiologist who was blinded to the BMI percentile groups. QTc was calculated using Bazett's correction formula (QTc = QT / √RR), and all QT and RR intervals were manually inspected to minimize potential inaccuracies from automated readings. ECG rhythm abnormalities were defined as the presence of any non‐sinus rhythm or rhythm irregularity, including sinus tachycardia, sinus arrhythmia, and isolated premature atrial or ventricular beats, as determined by the pediatric cardiologist.

### 2.4 Data Collection

Demographic data including age, sex, body weight, and height were recorded for all participants at baseline. Clinical characteristics and hemodynamic parameters (heart rate, systolic blood pressure, and diastolic blood pressure) were measured immediately before and at designated time points after the administration of methylphenidate. In addition, a standard 12‐lead electrocardiogram (ECG) was performed on each child both before and after methylphenidate administration. ECG parameters such as heart rate, PR interval, QRS duration, QT interval, and corrected QT interval (QTc) were evaluated. All data were documented using standardized data collection forms to ensure consistency and accuracy.

### 2.5 Statistical Analysis

All statistical analyses were performed using IBM SPSS Statistics version 27.0 (IBM Corp., Armonk, NY, USA). The normality of the data distribution was assessed using the Kolmogorov–Smirnov test. Continuous variables were presented as mean ± standard deviation, while categorical variables were expressed as numbers and percentages. Comparisons between pre‐treatment and post‐treatment measurements were performed using the paired samples *t*‐test for normally distributed variables and the Wilcoxon signed‐rank test for non‐normally distributed variables. Comparisons between groups according to BMI percentiles were conducted using the independent samples *t*‐test or the Mann–Whitney U test, as appropriate. Categorical variables were compared using the chi‐square test or Fisher's exact test. A *p* < 0.05 was considered statistically significant. *An* a priori *power analysis was performed using G*Power (version 3.1) for a two‐tailed paired *t*‐test comparing pre‐ and post‐treatment hemodynamic measurements. Assuming a medium effect size (Cohen's dz. = 0.5), an alpha level of 0.05, and a desired power of 0.95, the required sample size was calculated as 54 participants. In the present study, 60 children were included, indicating that the sample size was adequate to detect the expected effect.

## 3 Results

The mean age of the participants was 11.9 ± 2.2 years, and 66.7% of the group were male. The majority of the participants (80.0%) were within the 5th to 85th percentile BMI range. Prior to treatment, the mean systolic blood pressure was 105.0 ± 7.7 mmHg, and the mean diastolic blood pressure was 69.3 ± 9.2 mmHg. The average heart rate was 79.8 ± 9.0 bpm, and the mean peripheral oxygen saturation was 97.7% ± 1.3%. Pre‐treatment electrocardiographic measurements revealed that the mean QTc, PR, and QRS intervals were 395.9 ± 11.6 ms, 135.8 ± 16.8 ms, and 91.1 ± 9.5 ms, respectively. All participants had a normal ECG rhythm prior to treatment (Table 1).

**TABLE 1:** Demographic, anthropometric, and pre‐treatment (BT) hemodynamic characteristics of the participants.

Age (years)		11.9 ± 2.2
Sex	Female	20 (33.3)
	Male	40 (66.7)
BMI Group	5p‐85p	48 (80.0)
	< 5p	12 (20.0)
BMI Percentile		42.5 ± 28.3
BT SBP (mmHg)		105.0 ± 7.7
BT DBP (mmHg)		69.3 ± 9.2
BT Heart Rate (bpm)		79.8 ± 9.0
BT Peripheral SpO_2_ (%)		97.7 ± 1.3
BT QTc Interval (ms)		395.9 ± 11.6
BT PR Interval (ms)		135.8 ± 16.8
BT QRS Duration (ms)		91.1 ± 9.5
BT ECG Rhythm		60 (100)

*Note:* Data are presented as mean ± standard deviation for continuous variables and as number (%) for categorical variables.

Abbreviations: BMI, body mass index; BT, before treatment; DBP, diastolic blood pressure; SBP, systolic blood pressure; SpO_2_: Peripheral oxygen saturation.

After treatment, the mean systolic blood pressure was 110.1 ± 8.9 mmHg and the mean diastolic blood pressure was 73.4 ± 9.8 mmHg. The average heart rate was 84.9 ± 9.8 bpm, while the mean peripheral oxygen saturation was 98.3% ± 1.1%. Following treatment, the mean QTc, PR, and QRS intervals were measured as 401.0 ± 12.5 ms, 140.9 ± 16.7 ms, and 93.6 ± 9.5 ms, respectively. After treatment, 15.0% (*n* = 9) of the participants exhibited abnormal ECG rhythms, whereas 85.0% (*n* = 51) maintained a normal ECG rhythm (Table 2).

**TABLE 2:** Post‐treatment (after treatment; AT) hemodynamic and electrocardiographic characteristics of the participants.

AT SBP (mmHg)		110.1 ± 8.9
AT DBP (mmHg)		73.4 ± 9.8
AT Heart Rate (bpm)		84.9 ± 9.8
AT Peripheral SpO_2_ (%)		98.3 ± 1.1
AT QTc Interval (ms)		401.0 ± 12.5
AT PR Interval (ms)		140.9 ± 16.7
AT QRS Duration (ms)		93.6 ± 9.5
AT ECG Rhythm	Normal	51 (85.0)
	Abnormal	9 (15.0)

*Note:* Data are presented as mean ± standard deviation or number (percentage), as appropriate.

Abbreviations: AT, after treatment; DBP, diastolic blood pressure; ECG, electrocardiogram; PR, PR interval; QRS, QRS duration; QTc, corrected QT interval; SBP, systolic blood pressure; SpO_2_: peripheral oxygen saturation.

Following treatment, significant increases were observed in systolic blood pressure (from 105.0 ± 7.7 mmHg to 110.1 ± 8.9 mmHg, *p* < 0.001), diastolic blood pressure (from 69.3 ± 9.2 mmHg to 73.4 ± 9.8 mmHg, *p* < 0.001), heart rate (from 79.8 ± 9.0 bpm to 84.9 ± 9.8 bpm, *p* < 0.001), QTc interval (from 395.9 ± 11.6 ms to 401.0 ± 12.5 ms, *p* < 0.001), PR interval (from 135.8 ± 16.8 ms to 140.9 ± 16.7 ms, *p* < 0.001), and QRS duration (from 91.1 ± 9.5 ms to 93.6 ± 9.5 ms, *p* < 0.001). No significant change was observed in peripheral oxygen saturation (97.7% ± 1.3% before treatment vs. 98.3% ± 1.1% after treatment, *p* = 0.368). While all participants exhibited normal ECG rhythm prior to treatment, ECG rhythm abnormalities, including sinus tachycardia, sinus arrhythmia, and isolated premature atrial or ventricular beats, were observed in 15.0% (*n* = 9) of the participants after treatment (*p* = 0.002) (Table 3).

**TABLE 3:** Comparison of hemodynamic and electrocardiographic parameters of the participants before and after treatment.

		Before treatment	After treatment	*p*
SBP (mmHg)		105.0 ± 7.7	110.1 ± 8.9	**< 0.001**
DBP (mmHg)		69.3 ± 9.2	73.4 ± 9.8	**< 0.001**
Heart Rate (bpm)		79.8 ± 9.0	84.9 ± 9.8	**< 0.001**
Peripheral SpO_2_ (%)		97.7 ± 1.3	98.3 ± 1.1	0.368
QTc Interval (ms)		395.9 ± 11.6	401.0 ± 12.5	**< 0.001**
PR Interval (ms)		135.8 ± 16.8	140.9 ± 16.7	**< 0.001**
QRS Duration (ms)		91.1 ± 9.5	93.6 ± 9.5	**< 0.001**
ECG Rhythm	Normal	60 (100.0)	51 (85.0)	**0.002**
	Abnormal	0 (0.0)	9 (15.0)	

*Note:* Data are presented as mean ± standard deviation or number (percentage), as appropriate. Statistically significant *p* values are shown in bold.

Abbreviations: DBP, diastolic blood pressure; ECG, electrocardiogram; PR, PR interval; QRS, QRS duration; QTc, corrected QT interval; SBP, systolic blood pressure; SpO_2_, peripheral oxygen saturation.

A comparison of pre‐treatment hemodynamic and electrocardiographic parameters according to the participants' BMI percentile groups is presented in Table 4. The mean pre‐treatment systolic blood pressure was significantly lower in the < 5th percentile group compared to the 5th–85th percentile group (100.1 ± 7.1 mmHg vs. 106.3 ± 7.4 mmHg, *p* = 0.011). No significant differences were found between the groups with respect to diastolic blood pressure (67.7 ± 10.2 mmHg vs. 69.7 ± 9.0 mmHg, *p* = 0.493), heart rate (79.4 ± 8.4 bpm vs. 79.9 ± 9.2 bpm, *p* = 0.970), peripheral oxygen saturation (98.3% ± 1.4% vs. 97.6% ± 1.2%, *p* = 0.094), QTc interval (393.2 ± 10.1 ms vs. 396.6 ± 11.9 ms, *p* = 0.499), PR interval (144.1 ± 17.0 ms vs. 133.8 ± 16.2 ms, *p* = 0.063), or QRS duration (89.7 ± 8.9 ms vs. 91.4 ± 9.7 ms, *p* = 0.523) (Table 4).

**TABLE 4:** Comparison of pre‐treatment (BT) hemodynamic and electrocardiographic parameters according to body mass index (BMI) percentile groups of the participants.

	< 5p	5–85p	*p*
BT SBP (mmHg)	100.1 ± 7.1	106.3 ± 7.4	**0.011**
BT DBP (mmHg)	67.7 ± 10.2	69.7 ± 9.0	0.493
BT Heart Rate (bpm)	79.4 ± 8.4	79.9 ± 9.2	0.970
BT Peripheral SpO_2_ (%)	98.3 ± 1.4	97.6 ± 1.2	0.094
BT QTc Interval (ms)	393.2 ± 10.1	396.6 ± 11.9	0.499
BT PR Interval (ms)	144.1 ± 17.0	133.8 ± 16.2	0.063
BT QRS Duration (ms)	89.7 ± 8.9	91.4 ± 9.7	0.523

*Note:* Data are presented as mean ± standard deviation. Statistically significant *p* values are shown in bold.

Abbreviations: BMI, body mass index; BT, before treatment; DBP, diastolic blood pressure; PR, PR interval; QRS, QRS duration; QTc, corrected QT interval; SBP, systolic blood pressure; SpO_2_: peripheral oxygen saturation.

A comparison of post‐treatment hemodynamic and electrocardiographic parameters according to the participants' BMI percentile groups is shown in Table 5. The mean post‐treatment systolic blood pressure was significantly lower in the < 5th percentile group compared to the 5th–85th percentile group (104.4 ± 8.1 mmHg vs. 111.5 ± 8.6 mmHg, *p* = 0.011). The < 5th percentile group also had a significantly longer PR interval compared to the 5th–85th percentile group (148.4 ± 16.9 ms vs. 139.0 ± 16.3 ms, *p* = 0.038). There were no significant differences between the groups in terms of diastolic blood pressure (72.2 ± 10.4 mmHg vs. 73.7 ± 9.7 mmHg, *p* = 0.704), heart rate (83.8 ± 9.5 bpm vs. 85.2 ± 9.9 bpm, *p* = 0.535), peripheral oxygen saturation (98.0% ± 0.7% vs. 98.4% ± 1.2%, *p* = 0.328), QTc interval (397.5 ± 9.9 ms vs. 401.9 ± 13.0 ms, *p* = 0.380), or QRS duration (92.4 ± 9.0 ms vs. 93.9 ± 9.7 ms, *p* = 0.591). Following treatment, abnormal ECG rhythms were present in 75.0% (*n* = 9) of the < 5th percentile group, whereas all participants in the 5th–85th percentile group maintained a normal ECG rhythm (*p* < 0.001) (Table 5).

**TABLE 5:** Comparison of post‐treatment (AT) hemodynamic and electrocardiographic parameters according to body mass index (BMI) percentile groups of the participants.

		< 5p	5–85p	*p*
AT SBP (mmHg)		104.4 ± 8.1	111.5 ± 8.6	**0.011**
AT DBP (mmHg)		72.2 ± 10.4	73.7 ± 9.7	0.704
AT Heart Rate (bpm)		83.8 ± 9.5	85.2 ± 9.9	0.535
AT Peripheral SpO_2_ (%)		98.0 ± 0.7	98.4 ± 1.2	0.328
AT QTc Interval (ms)		397.5 ± 9.9	401.9 ± 13.0	0.380
AT PR Interval (ms)		148.4 ± 16.9	139.0 ± 16.3	**0.038**
AT QRS Duration (ms)		92.4 ± 9.0	93.9 ± 9.7	0.591
AT ECG Rhythm	Normal	3 (25.0)	48 (100.0)	**< 0.001**
	Abnormal	9 (75.0)	0 (0.0)	

*Note:* Data are presented as mean ± standard deviation or number (percentage), as appropriate. Statistically significant *p* values are shown in bold.

Abbreviations: AT, after treatment; BMI, body mass index; DBP, diastolic blood pressure; ECG, electrocardiogram; PR, PR interval; QRS, QRS duration; QTc, corrected QT interval; SBP, systolic blood pressure; SpO_2_, peripheral oxygen saturation.

No statistically significant differences were observed between female and male participants in baseline or post‐treatment hemodynamic and electrocardiographic parameters (all *p* > 0.05) (Table 6).

**TABLE 6:** Comparison of baseline and post‐treatment hemodynamic and electrocardiographic parameters according to sex.

Parameter	Female	Male	*p*
BT SBP (mmHg)	106.0 ± 8.5	104.5 ± 7.4	0.432
BT DBP (mmHg)	70.4 ± 9.2	68.8 ± 9.3	0.400
BT Heart Rate (bpm)	78.6 ± 8.5	80.5 ± 9.2	0.414
BT Peripheral SpO_2_ (%)	97.5 ± 1.5	97.9 ± 1.1	0.165
BT QTc Interval (ms)	396.8 ± 13.4	395.5 ± 10.7	0.814
BT PR Interval (ms)	135.2 ± 13.6	136.2 ± 18.3	0.894
BT QRS Duration (ms)	92.4 ± 8.0	90.4 ± 10.2	0.470
AT SBP (mmHg)	111.6 ± 9.6	109.4 ± 8.6	0.334
AT DBP (mmHg)	74.8 ± 9.7	72.7 ± 9.9	0.315
AT Heart Rate (bpm)	84.1 ± 9.0	85.3 ± 10.3	0.556
AT Peripheral SpO_2_ (%)	98.4 ± 1.1	98.3 ± 1.1	0.708
AT QTc Interval (ms)	402.3 ± 13.9	400.4 ± 11.9	0.643
AT PR Interval (ms)	140.7 ± 13.6	141.1 ± 18.2	0.869
AT QRS Duration (ms)	94.5 ± 7.9	93.2 ± 10.3	0.700

*Note:* Data are presented as mean ± standard deviation.

Abbreviations: AT, after treatment; BT, before treatment; DBP, diastolic blood pressure; QTc, corrected QT interval; SBP, systolic blood pressure.

## 4 Discussion

This study evaluated the effects of methylphenidate treatment on hemodynamic parameters in children diagnosed with attention‐deficit/hyperactivity disorder (ADHD). Our findings indicate that methylphenidate use leads to statistically significant changes in key hemodynamic and electrocardiographic parameters, particularly systolic and diastolic blood pressure, heart rate, QTc, and PR intervals. In addition, alterations in ECG rhythm were observed in some patients during the post‐treatment period. These results suggest that methylphenidate can exert notable physiological effects on the cardiovascular system, highlighting the importance of close hemodynamic monitoring during treatment in pediatric patients. In line with the current literature, our data further support the idea that methylphenidate can significantly influence both autonomic responses and cardiac conduction parameters.

In our study, methylphenidate treatment was associated with significant changes in systolic and diastolic blood pressure, heart rate, and ECG parameters in children. In contrast, a comprehensive meta‐analysis by Hennissen et al. (2017) [10] reported that methylphenidate therapy led to a significant increase only in systolic blood pressure among children and adolescents, with no statistically significant changes observed in diastolic blood pressure or heart rate. While both studies highlight the impact of methylphenidate on systolic blood pressure, the additional significant increases in diastolic blood pressure and heart rate observed in our cohort may be attributable to differences in patient characteristics, duration of treatment, or follow‐up protocols. Furthermore, although Hennissen et al. [10] did not identify clinically meaningful alterations in ECG parameters, our study found rhythm disturbances on ECG in a subset of patients following treatment, suggesting that methylphenidate may, albeit rarely, affect cardiac conduction. These findings underscore the clinical importance of close monitoring of hemodynamic and electrocardiographic parameters in children receiving methylphenidate therapy.

There is a limited body of research addressing the cardiac effects of MPH monotherapy in children diagnosed with ADHD [11, 12, 13]. In one study, Lamberti et al. [11] evaluated electrocardiographic changes in pediatric ADHD patients by measuring ECG parameters before and 2 h after administering a single 10 mg oral dose of MPH. Their findings indicated no significant alterations in T‐peak to T‐end interval (TpTe), QTc, or QT dispersion (QTd) values between baseline and post‐treatment measurements. However, there was a statistically significant rise in the TpTe/QTc ratio following MPH administration. Additionally, it has been reported that patients receiving medication (MPH, risperidone, or both) exhibited significantly increased TpTe interval, TpTe dispersion, and TpTe/QT ratio compared to both untreated ADHD patients and healthy controls [12]. Although QT interval duration has been associated with myocardial repolarization heterogeneity, it is not universally regarded as a dependable marker for predicting polymorphic ventricular tachycardia (torsades de pointes) or sudden cardiac death [14]. Since T‐wave morphology has been shown to play a predictive role in the development of drug‐induced polymorphic ventricular tachycardia, detailed evaluation and understanding of this parameter are essential [15, 16, 17]. Several investigations have suggested that the TpTe interval, TpTe dispersion, and TpTe/QT ratio may provide greater accuracy than the QT interval or QTd for forecasting ventricular arrhythmias. Consequently, these specific indices were analyzed in this study for their potential to offer superior predictive value [18, 19].

In the study conducted by Tanır et al. (2023), it was demonstrated that children receiving long‐acting methylphenidate exhibited significant increases in heart rate as well as systolic and diastolic blood pressure. Additionally, notable elevations in ventricular repolarization parameters—specifically TpTe, TpTe/QT, and TpTe/QTc—were observed on ECG [20]. Consistent with these findings, our study also revealed significant post‐treatment increases in cardiovascular and electrophysiological parameters, such as heart rate, systolic and diastolic blood pressure, QTc, and PR interval, following methylphenidate administration in children. Both studies indicate that methylphenidate can exert pronounced effects on cardiac conduction and ventricular repolarization. Similar to the findings of Tanır et al., our research also showed that methylphenidate may cause alterations in ECG markers associated with increased arrhythmic risk. However, while Tanır et al. reported that long‐term use of MPH did not result in additional adverse effects, our study found a higher incidence of arrhythmias in the post‐treatment period, particularly among children in the lower BMI group. These differences may be attributable to variations in patient demographics, duration of follow‐up, or the specific MPH formulation used. Overall, these findings further emphasize the clinical importance of regular monitoring of cardiovascular and electrophysiological parameters in children undergoing methylphenidate therapy.

Psychostimulant medications, notably methylphenidate (MPH), are considered the primary pharmacological intervention for ADHD, with class 1 evidence supporting their effectiveness [21, 22]. Despite their proven efficacy, concerns persist regarding their potential adverse effects. For example, a large‐scale retrospective study found no cardiac side effects associated with MPH use [23]. In contrast, a prospective investigation identified an increased incidence of arrhythmias (23%), cerebrovascular events (9%), and hypertension (8%) linked to these agents [24]. Nevertheless, research specifically focusing on the cardiovascular impacts—particularly the propensity for ventricular arrhythmias—of psychostimulants in pediatric populations remains scarce [11, 12, 13].

In the study by Şimşek et al. (2022), it was demonstrated that children with ADHD experienced significant increases in heart rate as well as systolic and diastolic blood pressure after 3 months of methylphenidate treatment [25]. Furthermore, marked elevations in ECG parameters such as Tp‐Te interval, Tp‐Te dispersion, and Tp‐Te/QTc ratio were observed, while QRS, QT, QTc, and QTdis values did not show significant changes. These findings suggest that methylphenidate therapy may enhance ventricular repolarization heterogeneity and, consequently, increase arrhythmia risk. Similarly, in our study, we observed significant post‐treatment increases in heart rate, systolic and diastolic blood pressure, as well as prolongation of the QTc and PR intervals, and the occurrence of ECG rhythm abnormalities following methylphenidate administration. Both studies highlight that methylphenidate can induce notable alterations in cardiovascular and electrophysiological parameters, emphasizing the importance of monitoring novel markers (such as Tp‐Te, Tp‐Te dispersion, and Tp‐Te/QTc ratio) that may indicate elevated arrhythmic risk. However, while Şimşek et al. did not observe changes in QTc and QTdis values, our study identified significant QTc prolongation and the development of ECG rhythm abnormalities in some cases. These discrepancies may be attributable to differences in the demographic characteristics of the study populations, treatment duration, or monitoring protocols. Ultimately, the present findings underscore the necessity of comprehensive and careful monitoring of hemodynamic and electrocardiographic parameters in children receiving methylphenidate therapy.

Although the observed changes in blood pressure, heart rate, and electrocardiographic parameters were statistically significant, the absolute magnitude of these changes was generally modest and is unlikely to translate into clinically significant consequences in otherwise healthy children. Importantly, these ECG rhythm abnormalities were predominantly transient and clinically non‐serious findings, and none required discontinuation of methylphenidate therapy. However, the occurrence of rhythm abnormalities, particularly among children with lower BMI percentiles, may indicate increased susceptibility in specific subgroups. According to current clinical recommendations, baseline cardiovascular evaluation prior to initiating stimulant treatment should primarily rely on a detailed medical history, family history, and physical examination, including measurement of blood pressure and pulse. Routine echocardiographic screening is not recommended in the absence of clinical suspicion, consistent with current guideline‐based practice. Further cardiac evaluation, including ECG or referral to cardiology, is advised only in patients with abnormal findings such as syncope, palpitations, abnormal physical examination, or a family history of sudden cardiac death [26].

The observation that even a low dose of 10 mg methylphenidate was associated with measurable cardiovascular changes suggests that these effects may potentially become more pronounced at higher doses. It should also be noted that a short‐acting formulation of methylphenidate was used in this study, which may result in more rapid peak plasma concentrations and make transient cardiovascular effects more readily detectable compared to long‐acting formulations. In contrast, long‐acting formulations provide a more gradual release profile, which may attenuate peak‐related physiological responses. Although previous studies have generally reported that the cardiovascular effects of methylphenidate are modest and clinically manageable, a potential dose–response relationship should be considered. Therefore, careful monitoring may be particularly important when higher doses are used in clinical practice. In this context, individualized dose titration remains essential, and the use of the lowest effective dose should be prioritized whenever possible. Furthermore, combining pharmacological treatment with behavioral and psychosocial interventions may enhance therapeutic outcomes while potentially reducing the need for higher medication doses, thereby minimizing the risk of adverse effects.

This study has several limitations. It was conducted at a single center with a relatively small sample size, which may limit the generalizability of the findings. The pediatric cardiologist was blinded to the BMI percentile groups during ECG evaluation; therefore, observer‐related bias cannot be completely excluded. The study did not include a separate untreated control group or a placebo condition. Although the pre–post design allowed within‐subject comparison during treatment initiation, causal inference regarding the observed acute ECG and hemodynamic changes remains limited. The study design focused exclusively on the acute peak‐effect period following a single 10 mg dose of short‐acting methylphenidate. Therefore, cumulative cardiovascular effects, adaptive physiological responses that may occur with daily long‐term treatment, and dose‐escalation‐related risks could not be assessed. Although this limits the ability to infer long‐term safety, evaluating early hemodynamic and electrophysiological changes during treatment initiation is clinically relevant, as adverse responses often emerge at the onset of therapy. All participants were free of known cardiovascular disease and concomitant medication use; thus, the findings may not be directly applicable to children with comorbidities or those receiving multiple medications. In addition, sex‐related differences in ECG parameters, particularly QTc, may emerge during late childhood and adolescence. Although sex‐stratified analyses were performed, the present sample size was modest and the study was not specifically powered to detect subtle sex‐specific effects. Additionally, assessments of ventricular repolarization were limited to standard 12‐lead ECG recordings, without Holter monitoring or advanced electrophysiological testing, which may have resulted in underdetection of transient arrhythmic events. Given these limitations, the present findings should be interpreted with caution and confirmed by multicenter studies with larger cohorts, more comprehensive monitoring methods, and longer follow‐up periods.

In this study, it was demonstrated that methylphenidate treatment in children with ADHD leads to statistically significant increases in systolic and diastolic blood pressure, heart rate, and certain electrocardiographic parameters such as QTc and PR interval. In addition, some patients developed abnormal ECG rhythms after treatment. These findings suggest that methylphenidate has noticeable effects on both hemodynamic and cardiac conduction parameters. Therefore, close monitoring of cardiovascular and electrocardiographic parameters is recommended during methylphenidate treatment, especially in children with additional risk factors. Further studies with larger sample sizes and longer follow‐up periods are needed to more clearly establish the long‐term safety profile of methylphenidate in the pediatric population.

## Author Contributions

C.C.D.J. contributed to the conception and design of the study, patient recruitment, data collection, data interpretation, and drafting of the manuscript. S.V.Y. contributed to the study methodology, cardiologic evaluation and electrocardiographic interpretation, data interpretation, and critical revision of the manuscript. Both authors reviewed and approved the final version of the manuscript and agree to be accountable for all aspects of the work.

## Funding

The authors have nothing to report.

## Conflicts of Interest

The authors declare no conflicts of interest.

## Data Availability

The data that support the findings of this study are available from the corresponding author upon reasonable request.
